# Using social practice theory in measuring perceived stigma among female sex workers in Mombasa, Kenya

**DOI:** 10.1186/s12889-023-15809-2

**Published:** 2023-05-26

**Authors:** Joseph Newton Guni, Stanley Wechuli Wanjala, Griffins Manguro, Caroline Gichuki, Megan SC Lim, Minh D. Pham, Stanley Luchters, James Orwa

**Affiliations:** 1 Institute for Human Development, Aga Khan University, Nairobi, Kenya; 2 Department of Public Health and Primary Care, Faculty of Medicine and Health Sciences, Ghent University, Ghent, Belgium; 3 Department of Social Sciences, School of Humanities and Social Sciences, Pwani University, Kilifi, Kenya; 4 Department of Population Health, Aga Khan University, Nairobi, Kenya; 5 Centre for Sexual Health and HIV AIDS Research, CeSHHAR, Harare, Zimbabwe; 6 Liverpool School of Tropical Medicine, Liverpool, UK; 7 Burnet Institute, Melbourne, Australia; 8 School of Population Health and Preventive Medicine, Monash University, Melbourne, Australia; 9 Melbourne school of global and population health, University of Melbourne, Parkville, Australia; 10 Department of Epidemiology and Preventative Medicine, Monash University, Melbourne, Australia; 11 International Center for Reproductive Health, Mombasa, Kenya; 12 Institute for Human Development, Aga Khan University, Nairobi, Kenya

**Keywords:** Female sex workers, Perceived stigma, Index, Social practice theory, Factor analysis, Kenya

## Abstract

**Background:**

Perceived stigma is a complex societal phenomenon that is harboured especially by female sex workers because of the interplay of a myriad of factors. As such, a precise measure of the contribution of different social practices and characteristics is necessary for both understanding and intervening in matters related to perceived stigma. We developed a Perceived Stigma Index that measures the factors that greatly contribute to the stigma among sex workers in Kenya, and thereby inform a framework for future interventions.

**Methods:**

Social Practice Theory was adopted in the development of the Perceived Stigma Index in which three social domains were extracted from data collected in the WHISPER or SHOUT study conducted among female sex workers (FSW), aged 16–35 years in Mombasa, Kenya. The three domains included: Social demographics, Relationship Control and Sexual and Gender-based Violence, and Society awareness of sexual and reproductive history. The factor assessment entailed Exploratory Factor Analysis (EFA), Confirmatory Factor Analysis (CFA), and the internal consistency of the index was measured using Cronbach’s alpha coefficient.

**Results:**

We developed a perceived stigma index to measure perceived stigma among 882 FSWs with a median age of 26 years. A Cronbach’s alpha coefficient of 0.86 (95% confidence interval (CI) 0.85–0.88) was obtained as a measure of the internal consistency of our index using the Social Practice Theory. In regression analysis, we identified three major factors that contribute to the perceived stigma and consists of : (i) income and family support (β = 1.69; 95% CI); (ii) society’s awareness of the sex workers’ sexual and reproductive history (β = 3.54; 95% CI); and (iii) different forms of relationship control e.g. physical abuse (β = 1.48; 95%CI that propagate the perceived stigma among the FSWs.

**Conclusion:**

Social practice theory has solid properties that support and capture the multi-dimensional nature of perceived stigma. The findings support the fact that social practices contribute or provoke this fear of being discriminated against. Thus, in offering interventions to curb perceived stigma, focus should fall on the education of the society on the importance of acceptance and integration of the FSWs as part of the society and the eradication of sexual and gender based violence meted out on them.

**Trial registration:**

The trial was registered in the Australian New Zealand Clinical Trials Registry, ACTRN12616000852459.

## Introduction

Stigma is a complex societal phenomenon [1–3]. Despite the increasing interest and understanding of the adverse health outcomes associated with stigma [4], there is an exigent need of narrowing down and highlighting the factor-specific societal contribution to stigma. These factors, i.e., societal beliefs and practices, play a major role in creating the perceived stigma which in essence is the fear of being discriminated against or the fear of enacted stigma where the stigmatized persons internalize prejudices and develop negative feelings about themselves [5]. Consequently, they end up feeling abashed and embarrassed about the practice [6]. On a larger scale, this perceived stigma has the potential of curtailing societal advancements in terms of academic achievements and decreasing the uptake of health and social services [7].

While many studies are being conducted among sex workers, they have extensively sought to delve into HIV, Sexually Transmitted Infections (STIs) and some behavioral risk factors like Sexual and Gender-Based Violence [8–11]. Many of these studies have also concentrated on HIV/AIDS-associated stigma as a major encumbrance to voluntary testing, counseling and the necessary prompt treatment [12, 13]. However, just a few studies have endeavored to scrutinize and provide a measure of the perceived stigma related to being a sex worker and identify the key variables of stigmatizing sources [14, 15].

Perceived stigma is usually related, contributed and exacerbated by society. This is profoundly in relation to the expected reactions towards the activities of these sex workers [16]. In most circumstances, these sex workers are predisposed to a plethora of stigmatizing forces in their daily lives through their interactions with relatives, neighbors, religious institutions, health providers and law enforcers. These societal prejudices may have deleterious effects on the health and well-being of the sex workers through obvious manifestations such as physical or verbal abuse and through slicker and subtle means such as those that propagate and immortalize vulnerability which then obliges these workers to initiate personal individualized mechanisms or collective ways of dealing with the stigma [17].

In some cases, this stigmatization is due to how sex work is perceived in the legal sphere. The different legal systems have advanced for either the full or partial criminalization of the trade. Interestingly, the partial criminalization creates a disconnect between society and sex workers, thus breeding the perceived stigma [18–20]. In the Kenyan context, such partial criminalization of sex work also exists [21, 22]. This disjointed relationship is often exhibited by denigratory labels on the female sex workers such as ‘prostitutes’, ‘hookers’, and ‘whores’ in describing the sex work [18].

A significant body of research has shown that female sex workers experience higher levels of stigma and related violence compared to Male Sex Workers [23]. However, few studies have endeavored to provide a measure of this perceived stigma among female sex workers. This paper aims to establish a thorough factor-structured index verifiable in exploratory and confirmatory factor analyses with acceptable goodness of fit of the societal contributory nature to the perceived stigma among sex workers.

The few developed Perceived Stigma Indices among FSWs [24–26] have all adopted different concepts and structures in their measurements of stigma. This entailed using factors pooled from bases that are sometimes different and peculiar to certain societies. As envisaged in previous research on stigma measurement, indices from different populations and settings can only be used in the scale development and initial validation; however, they cannot be equated to every new society. As unequivocally stated in the USAID 2006 report: “Constructing a Stigma and Discrimination Index: Hopes, Dreams, and Lessons Learned,” no standardized measure or index can be adopted to encompass all the relevant variables and factors in different settings.

Our analysis aimed to develop a standardized instrument among the female sex workers Perceived Stigma Index (PSI) to quantify stigma and measure the contributory capacity of the societal factors; and also to identify the key components of stigmatizing sources under the Social Practice Theory that will allow for tracking the levels of the Perceived Stigma and thus provide a mechanism for reduction interventions. This secondary analysis was conducted from the WHISPER or SHOUT study that was conducted among female sex workers in Mombasa, Kenya [27, 28].

## Methodology

This paper describes a secondary analysis of the data from the WHISPER or SHOUT study: Women’s Health Intervention using SMS for Preventing Unintended Pregnancy (WHISPER) and SMS intervention to improve Nutritional Health Outcomes (SHOUT) study. The study methods are described in detail elsewhere [27, 28].

## Social practice theory

Social Practice Theory offers a platform to understand the similitude and connection between the societal practices and stigma among female sex workers. Social practice refers to the typical and habitual everyday practices performed in society [29]. For the purpose of this paper, social practice theory is defined as a theory that calls out how people pursue diverse concerns, become aware of new possibilities for action as they move across different settings of practice; and learn as they adjust contributions to the flow of the ongoing activity and to fit the demands and structures of local institutions [30]. It seeks to understand and explain the social and cultural world by analyzing the basic bodily, knowledge-based practices that interconnect to form more complex social entities like groups’ lifestyles, social fields or entire societies [29].

SPTs have been considered important in offering alternative yet concrete ways of understanding human action in relation to health and well-being; and to further reconcile structure and urgency in the lived experience of everyday life. Health and several aspects of well-being are considered the outcome of participation in a set of Social Practices. Social Practice Theory hypothesizes that better designed and managed neighborhoods recruit residents into new practices or reconfigure the existing ones, resulting in observed increases in health and well-being [31].

**Fig. 1:**
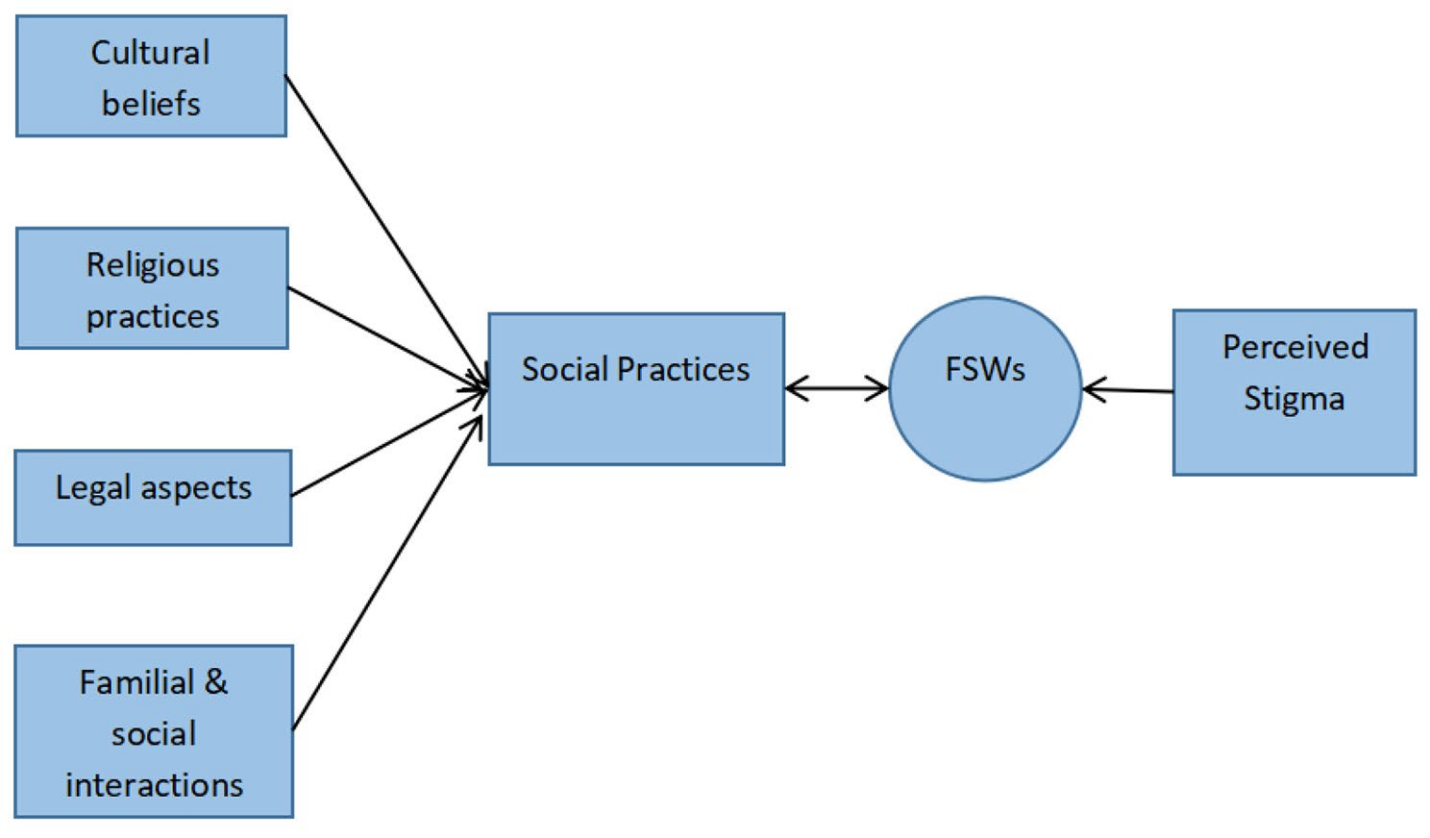
Conceptual framework on the connection between societal practices and perceived stigma among female sex workers

Thus through social practices, this paper seeks to understand how the internalized stigma among the female sex workers is generated. Figure 1 below conceptualizes how FSWs, in the full awareness of the different social practices, perceive all the prejudices and discrimination against them, and as such develop new mechanisms or behaviours to cope in the society.

## Index development

Several steps were taken to develop the female sex workers’ Perceived Stigma Index (PSI). A thorough examination of the availability of existing stigma measures did not result in identifying an appropriate tool, further laying credence to the minimum or non-existing research work in this area.

**Fig. 2:**
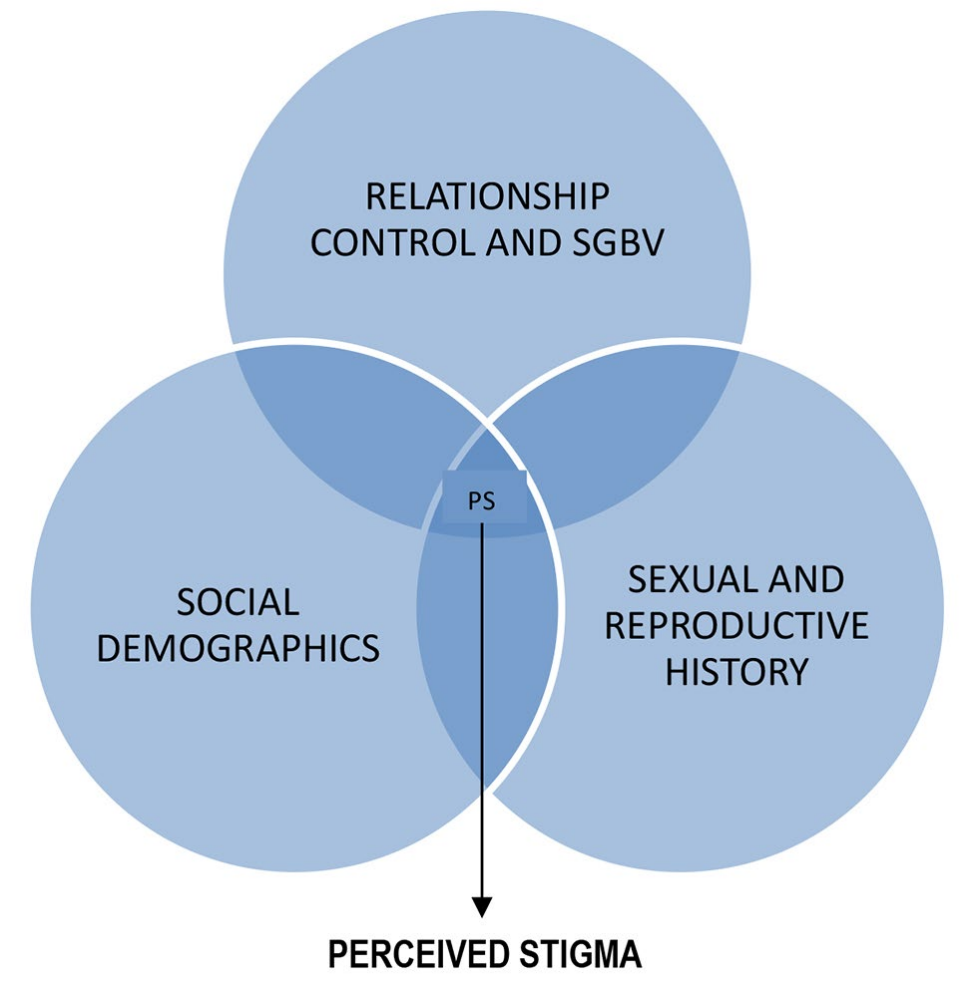
Interaction of societal factor domains

Three main factor domains that contribute significantly to the Perceived Stigma were identified from the research questionnaire used during the primary research (Fig. 2). The index was designed to capture factors whose interplay was deemed to contribute to the perceived stigma, and included: (1) Social demographic factors, (2) Relationship control & sexual and gender-based violence, and (3) Sexual and reproductive history and social awareness.

## Data analysis

### Factor analysis

In developing the female sex workers’ Perceived Stigma Index (PSI), the study employed various Structural Equation Modelling methods. A thorough and comprehensive analysis was conducted to interrogate the PSI’s reliability and validity. The internal consistency (how closely related the set of items are) of the Index and of the three domains therein was measured by the Cronbach’s alpha coefficients. Item Score Reliability test was used to assess the repeatability of an individual item score in the three groups. In this test, an item was deleted if its Item-rest correlation coefficient was lower than 0.40. At the end of the process, all the items in the three domains had Item-rest correlation coefficients higher than 0.40. This was very crucial for the purpose of satisfying the criteria of construct validity.

Primary Component Analysis, a subset of Exploratory Factor Analysis (EFA), was employed to verify the dimensionality of the index items. Other tools that include but are not limited to the Scree Plot Test, Parallel Analysis, Residual Variance, Cumulative Variance and Oblique Rotation were also performed as part of the EFA. This EFA was performed using R and subsequent Confirmatory Factor Analysis (CFA) was similarly done through R to verify the results.

The three-factor domains as envisaged from the Social Practice Theory were validated to be sufficient in providing a standard measure for the perceived Stigma among Female Sex Workers. Item endorsement was determined by calculating the median and the Inter-quartile ranges (IQRs) of the responses from each item. The scores for each scale were calculated separately by summing each weighted item in the index, with the weights being the factor loadings obtained from the Confirmatory Factor Analysis (CFA).

### Regression analysis

A descriptive analysis was conducted on the three social domains conjured through the Social Practice Theory regarding the perceived stigma. The proceeding Linear Regression Analysis was conducted using R on each domain separately. The weighted scores on each scale were used to determine how the influence of each of the Social Practice components correlates and contributes to the perceived stigma levels from the three sources.

## Results

### Social practice domains

The study enrolled 882 Female Sex Workers, who had a median age of 26 years (IQR: 22–29; Table 1). Participating women were predominantly born in Kenya (98%) with 77.2% having changed residences in the last two years of the baseline study. Majority of the women (72.3%) were single, with only 6.2% either married or living with a partner. The sex workers supported a median of 3 people financially (IQR:1–4), with their main source of income being sex work (99.2%). Most of them (99.2%) relied on an income from the trade with most receiving a payment after sex with a client of $ 5 and above (54.9%) in a period of six months.

Relationship control and instances of sexual and gender based violence (Table 2) are clearly highlighted by the respondents; from unilateral decision making (54.5%), whether or not they should use a condom (32.9%), control of what the females should wear (44.2%) and whom they spend time with (44.5%).

Sexual and reproductive history of the participants in relation to the societal awareness (Table 3) indicate the non-willingness of disclosure by the female sex workers of their trade to neither their spouses (40%) nor their parents or siblings (71.9%).

**Table 1:** Socio-Demographic characteristics of 882 female sex workers enrolled in the WHISPER or SHOUT study between 2016 and 2017 in Mombasa, Kenya, N = 882

Socio-Demographic variables	
**Age**, years	
Median (IQR)	26 (22–29)
**Country born** *n (%)*	
Kenya	864 (98.2)
Other East African country	16 (1.8)
**Have changed residence in the last two years** *n (%)*	
Yes	681 (77.8)
**Marital status** *n (%)*	
Legally/ formally married	30 (3.4)
Living with a partner/ boyfriend	25 (2.8)
Single (not living with a partner)	638 (72.3)
Separated/ divorced	176 (20.0)
Widowed	12 (1.4)
**Highest level of education** *n (%)*	
None	2 (0.2)
Not completed primary school	102 (11.6)
Completed primary school	257 (29.1)
Not completed secondary school	207 (23.5)
Completed secondary school	261 (29.6)
Completed tertiary training	52 (5.9)
**Religion** *n (%)*	
Protestant	395 (44.8)
Catholic	310 (35.1)
Muslim	172 (19.5)
Others	5 (0.6)
**Number of people supported financially**	
Median (IQR, range)	3 (1–4; 0–22)
***Sources of income in the last six months***	
Sex work (yes) n (%)	875 (99.2)
Petty commerce (yes) n (%)	113 (12.8)
Formal employment (yes) n (%)	18 (2.0)
Casual labor (yes) n (%)	9 (1.0)
**Average weekly income from sex work alone in the last six months** *n (%)*	
Less than $ 10	146 (16.6)
$ 10-$ 20	215 (24.4)
More than $20	517 (58.6)
**Average payment received after sex with client in the last six months** *n (%)*	
Less than $ 2	141 (16.0)
$ 2 - $ 4.99	255 (28.9)
$ 5 & above	484 (54.9)
**Average weekly income from other sources in the last six months** *n (%)*	
$ 0 - $ 10	167 (18.9)
$ 10.01- $ 20	95 (10.8)
$ 20.01 & above	614 (69.6)
No other income	6 (0.7)

**Table 2:** Shows the different forms of relationship control and instances of sexual and gender-based violence experienced by the female sex workers, N = 717

Relationship control and Sexual and Gender-Based Violence questions	n (%)
Whether boyfriend/ husband will beat me if I asked to use a condom	
Strongly agree	27 (3.7)
Agree	111 (15.4)
Disagree	442 (61.6)
Strongly disagree	137 (19.1)
Whether boyfriend/ husband will get angry if asked to use a condom	
Strongly agree	79 (11.0)
Agree	236 (32.9)
Disagree	342 (47.6)
Strongly disagree	60 (8.4)
Boyfriend/ husband won’t let me wear certain things	
Strongly agree	21 (2.9)
Agree	317 (44.2)
Disagree	332 (46.3)
Strongly disagree	45 (6.3)
Decisions are undertaken solely by boyfriend/ husband	
Strongly agree	50 (7.0)
Agree	391 (54.5)
Disagree	246 (34.3)
Strongly disagree	29 (4.0)
Boyfriend/ husband decides who I spend time with	
Strongly agree	37 (5.2)
Agree	319 (44.5)
Disagree	317 (44.2)
Strongly disagree	42 (5.9)
Boyfriend/ husband will think I am having sex with other people if I ask him to use a condom	
Strongly agree	84 (11.7)
Agree	295 (41.1)
Disagree	291 (40.6)
Strongly disagree	46 (6.4)
Feel trapped or stuck in my relationship	
Strongly agree	29 (4.0)
Agree	247 (34.4)
Disagree	404 (56.3)
Strongly disagree	35 (4.9)
Boyfriend/ husband always has his way with me	
Strongly agree	33 (4.6)
Agree	269 (37.5)
Disagree	387 (54.0)
Strongly disagree	29 (4.0)
Boyfriend/ husband gets his way even in disagreements	
Strongly agree	30 (4.2)
Agree	361 (50.3)
Disagree	305 (42.5)
Strongly disagree	19 (2.6)
Boyfriend/ husband always wants to know my whereabouts	
Strongly agree	71 (9.9)
Agree	458 (63.9)
Disagree	168 (23.4)
Strongly disagree	18 (2.5)
Boyfriend/ husband is having sex with other people	
trongly agree	62 (8.6)
Agree	285 (39.7)
Disagree	339 (47.3)
Strongly disagree	31 (4.3)
I have a good relationship with boyfriend/ husband	
Strongly agree	38 (5.3)
Agree	490 (68.3)
Disagree	158 (22.0)
Strongly disagree	31 (4.3)
Whether any partner ever pushed/ shoved you (yes)	435 (60.7)
Whether any partner ever slapped or thrown something hurtful at you (yes)	378 (52.7)
Whether partner hit you with a fist, kicked you or hit you with something else (yes)	251 (35.0)
Whether anyone ever physically forced you to have sex (yes)	341 (47.6)
Whether you have been sexually abused in the past 12 months (yes)	253 (35.3)
Whether you had sex with a partner because of fear (yes)	325 (45.3)
Whether any partner degraded or humiliated you sexually (yes)	224 (31.2)

**Table 3:** Shows the sexual and reproductive history of the participants in relation to the societal awareness of the same

Age when started sex work	
Median years (IQR)	20 (18–32)
No. of clients in the last seven days	
Median (IQR)	4 (3–6)
Use of condom when with a client *n (%)*	
Always	671 (76.1)
Most of the time	69 (7.8)
Sometimes	129 (14.6)
Never	7 (0.8)
*Missing*	6 (0.7)
Currently have a boyfriend or husband (yes)	495 (56.1)
No. of boyfriends/ husbands I have had sex with in the last seven days (Median (IQR))	1 (1–6)
Use of condom when having sex with boyfriends/ husbands *n (%)*	
Always	158 (17.9)
Most of the time	23 (2.6)
Sometimes	106 (12)
Never	200 (22.7)
*Missing / don’t have boyfriend or husband*	395 (44.8)
Awareness of boyfriends/ husbands that I am a sex worker *n (%)*	
Yes	138 (15.6)
No	353 (40)
Don’t know	3 (0.3)
*Missing / don’t have boyfriend or husband*	388 (44.0)
Awareness of parents and/or siblings that I am a sex worker *n (%)*	
Yes	96 (10.9)
Some but not all	144 (16.3)
No	634 (71.9)
Don’t know	6 (0.7)
*Missing*	2 (0.2)

### Item reduction and index reliability

The reliability of the Perceived Stigma Index was high, with a Cronbach’s alpha coefficient of 0.86 (Table 4). All the factors in the three domains satisfied the item-rest correlation of 0.4 and above, and so they were all retained in the perceived stigma index. The results from the primary component analysis, parallel analysis, and Screeplots laid credence to the social practice theory approach. We observed that our Social Practice Theory Approach only accounted for the 40% explained variance (Table 5). This illustrates the multi-dimensional construct of the perceived stigma .

**Table 4:** Internal consistency of items in Perceived stigma: item statement, corrected item-related correlation.alpha of deleted item and factor loadings (CFA).

Item No.	Item Statement	Corrected item-total correlation	Alpha of item deleted	Factor loadings (CFA)
**I feel that if I disclosed being a sex worker to….**				
1.	Some people, they would not talk to me anymore	0.67	0.85	0.67
2.	Some people, they would not talk to my family	0.61	0.85	0.60
3.	Some people, they would think I was immoral	0.67	0.85	0.68
4.	Some people, I would be threatened with violence	0.54	0.86	0.53
5.	Some people, they would treat me differently	0.65	0.85	0.65
6.	My husband/boyfriend, he would hit me	0.53	0.86	0.49
7.	My husband/boyfriend, he would not talk to me anymore	0.62	0.85	0.60
8.	My family, I would not be able to see my children	0.58	0.85	0.57
9	My family, they would desert me	0.73	0.84	0.72
10.	My family, they would treat me differently	0.71	0.84	0.72
	**Overall alpha (95% CI)**	0.86 (0.85–0.88)		

### Exploratory factor analysis

**Fig. 3:**
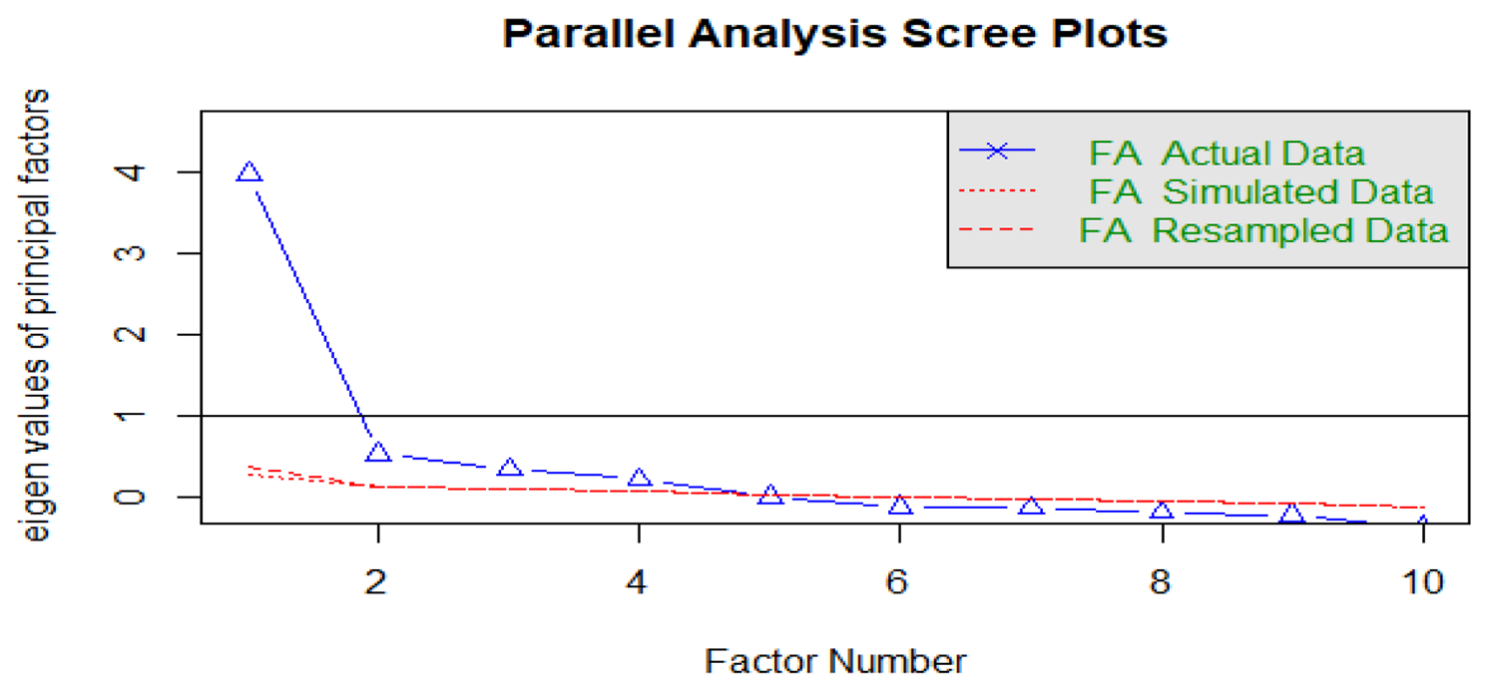
Scree plot from the EFA analysis

We performed a parallel analysis (maximum likelihood) using a polychoric correlation matrix which suggested that the social practice theory structure had only one factor with an eigen- value > 1.0 (Fig. 3) which also accounted for 40% of the variance.

**Table 5:** Shows the descriptive statistics for the Perceived stigms: Item statement and factor coefficients

Item	Item Statement	Factor coefficients (EFA)
**I feel that if I disclosed being a sex worker to….**		
1.	Some people, they would not talk to me anymore	0.67
2.	Some people, they would not talk to my family	0.61
3.	Some people, they would think I was immoral	0.68
4.	Some people, I would be threatened with violence	0.53
5.	Some people, they would treat me differently	0.65
6.	My husband/boyfriend, he would hit me	0.50
7.	My husband/boyfriend, he would not talk to me anymore	0.60
8.	My family, I would not be able to see my children	0.58
9	My family, they would desert me	0.73
10.	My family, they would treat me differently	0.72

Explained variance proportion is 40%

### Regression analysis

Tables 6, 7 and 8 indicate the relationships between the Female Sex workers’ perceived stigma and the three societal domains as envisaged through the social practice theory. The key results from the regression analysis indicate highest correlation between perceived stigma and income, relationship control and societal awareness; factors derived from the three domains.The results also show that the personal characteristics of the female sex worker did not contribute much to the perceived stigma compared to the other two domains.

**Table 6:** A multi variable linear model showing the relationships between socio-demographic predictor variables and Female Sex Workers Perceived stigma

Socio-Demographic	coefficient (95% CI)	P-value
**Age**	0.06 (-0.02,0.13)	0.123
**Religion**		
Protestant *(Reference)*		
Catholic	-0.92 (-1.69,-0.16)	0.018
Muslim	-1.18 (-2.1,-0.25)	0.013
Others	-6.23 (-10.74,-1.71)	0.007
**Average weekly income from sex work alone in the last six months**		
Less than $ 10 *(Reference)*		
$ 10.01-$ 20	0.95 (-0.19,2.09)	0.101
$ 20.01 & above	1.69 (0.59,2.78)	0.003
**Average payment received after sex with client in the last six months**		
Less than $ 2 *(Reference)*		
$0.2. 01-$ 4.99	-1.42 (-2.53,-0.31)	0.012
$ 5 & above	-2.12 (-3.24,-1.00)	< 0.001

**Table 7:** A multi-variable linear model showing the relationships between the predictor variables (Relationship control and Sexual and Gender-Based Violence) and female sex workers’ perceived stigma

	ß (95% CI)	P-value
Whether boyfriend/ husband will get angry if asked to use a condom		
Strongly agree *(Reference)*		
Agree	-1.5 (-2.8,-0.19)	0.025
Disagree	-1.14 (-2.57,0.28)	0.117
Strongly disagree	-0.55 (-2.52,1.42)	0.585
Boyfriend/ husband won’t let me wear certain things		
Strongly agree *(Reference)*		
Agree	-0.17 (-2.27,1.92)	0.871
Disagree	-1.5 (-3.58,0.59)	0.160
Strongly disagree	-3.48 (-6.02,-0.93)	0.008
Boyfriend/ husband will think I am having sex with other people if I ask him to use a condom		
Strongly agree *(Reference)*		
Agree	-1.59 (-2.88,-0.29)	0.017
Disagree	-2.64 (-4.12,-1.16)	< 0.001
Strongly disagree	-4.41 (-6.5,-2.32)	< 0.001
Feel trapped or stuck in my relationship		
Strongly agree *(Reference)*		
Agree	0.81 (-1.19,2.81)	0.428
Disagree	-0.22 (-2.21,1.78)	0.832
Strongly disagree	-2.89 (-5.48,-0.29)	0.029
Boyfriend/ husband gets his way even in disagreements		
Strongly agree *(Reference)*		
Agree	-2.02 (-3.82,-0.23)	0.028
Disagree	-2.29 (-4.1,-0.48)	0.013
Strongly disagree	-1.2 (-3.97,1.57)	0.397
Boyfriend/ husband is having sex with other people		
Strongly agree *(Reference)*		
Agree	-1.75 (-3.08,-0.42)	0.010
Disagree	-1.39 (-2.74,-0.05)	0.043
Strongly disagree	-2.09 (-4.22,0.03)	0.054
*Missing*		
I have a good relationship with boyfriend/ husband		
Strongly agree *(Reference)*		
Agree	-2.1 (-3.71,-0.49)	0.011
Disagree	-2.8 (-4.54,-1.06)	0.002
Strongly disagree	-2.22 (-4.45,0.02)	0.052
Whether any partner ever pushed/ shoved you		
No *(Reference)*		
Yes	0.68 (-0.12,1.48)	0.094
Whether partner hit you with a fist, kicked you or hit you with something else		
No *(Reference)*		
Yes	-0.54 (-1.41,0.33)	0.224
Whether anyone ever physically forced you to have sex		
No *(Reference)*		
Yes	1.48 (0.66,2.31)	< 0.001
Whether you had sex with a partner because of fear		
No *(Reference)*		
Yes	0.94 (0.1,1.77)	0.029

**Table 8:** A multi-variable linear model showing the relationships between the predictor variables (Sexual and reproductive history of the participants & societal awareness) and Female Sex Workers Perceived stigma

	ß (Std.Err)	P-value
Use of condom when with a client		
Always *(Reference)*		
Most of the time	1.64 (0.01,3.27)	0.049
Sometimes	3.3 (-2.25,8.84)	0.245
Never	0.84 (-0.39,2.07)	0.183
No. of boyfriends/ husbands I have had sex with in the last seven days	0.65 (-0.05,1.36)	0.070
Awareness of parents and/or siblings that I am a sex worker		
Yes *(Reference)*		
No	3.54 (2.57,4.51)	< 0.001
Don’t know	-2.46 (-8.04,3.13)	0.389

## Discussion

Our study aimed to develop a standardized instrument i.e., the PSI, to measure perceived stigma among female sex workers in Mombasa, Kenya. There being a very limited number of studies done in this space and context, and no comparable tool to quantify the perceptions of FSWs in the region, we endeavored to further consider the appraisal of specific factors that contribute to such perceptions. We adopted the Social Practice Theory by coming up with three societal domains that in some way were deemed to induce the perceived stigma. The factors from the three domains were subjected to both exploratory and confirmatory factor analyses and the multi-dimensional build-up of perceived stigma was proven. Linear regression models further provided a more distinctive and clear answer to the significant factors that contribute inordinately to perceived stigma among female sex workers. These major factors include: Income, Relationship Control, Sexual and Gender based violence and Parental and/ or siblings awareness of the person’s involvement in sex work.

### An increase in income and family support

We observed that an increase in income correlated to perceived stigma among the FSWs. An increase from the weekly income range of $ 10 – $ 20 to $ 20 and above created a significant increase in the PSI (0.95 to 1.69 change in the regression coefficients). This was despite the fact that the female sex workers had a median of 3 people they supported financially. This can simply be connected to another factor, i.e., societal awareness, where an increase in income would mean increased financial support to family members hence risking the knowledge of their sexual and reproductive history. In this case, financial stability or independence influences how women internalize the experienced perceived stigma [32].

### Societal awareness of the sexual activities

This poses the greatest contribution to perceived stigma among the aforementioned significant factors. A substantial 71.9% of the participants indicated that their parents and/or siblings were not aware that they were sex workers, and a further regression generated a very high correlation coefficient (3.54).

### Relationship control

The FSWs indicated that their husbands or partners had some form of control over their mode of dressing and decision-making. This, in turn, enacted the fear among the FSWs that their societal rights to be involved in such a trade weren’t in their control. This further informs the fact that a majority of the FSWs are single (72.3%); perhaps choosing to maintain their freedom that is unhinged from any kind of control by potential or prospective partners.

### Sexual and gender-based violence (SGBV)

The FSWs had experienced or observed some form of physical or sexual violence. Such dehumanizing acts had left an indelible mark on them, hence the high perceived stigma. These findings are in line with studies that have shown violence against the FSWs by either the partners or clients provokes this internalized stigma that has direct connections to suicidal thoughts and other depressive symptoms [33–35]. The violence meted against them was both sexual and physical in which the latter entailed the partners using kicks, fists and other objects on the FSWs.

## Conclusion

The developed PSI takes cognizance of the social practices in the community, which can be particularly useful in providing the requisite interventions and tracking the level of perceived stigma in different societies. This study provides the first instrument through which perceived stigma can be measured and controlled by only focusing on key societal variables: income and family support, societal awareness of the FSW and their trade, relationship control, and sexual and gender-based violence.

### Strengths and limitations of this study

The study’s strengths are that it was based on a large study population that provided a large data set that was really important for the psychometric analysis. Rigorous analytical tools were also employed in the measurement of invariance, an important aspect of structural validity. However, there were several limitations to this study. Out of the entire population that had been enrolled in the primary WHISPER or SHOUT study, 11% faced communication challenges in terms of network and language problems hence their data was not captured. This data would have offered more analytical base in the secondary analysis. Additionally, the 40% invariance indicates the need for further induction of other factors contributing to the perceived stigma.

## Data Availability

Study data are available from the corresponding author upon reasonable request.

## Abbreviations

WHISPER: Women’s Health Intervention Using SMS for Preventing Unintended Pregnancy
SHOUT: SMS Intervention to Improve Nutritional Health Outcomes
EFA: Exploratory factor analysis
CFA: Confirmatory factor analysis
PSI: Perceived Stigma Index
STIs: Sexually Transmitted Infections
FSWs: Female Sex Workers
HIV/AID: Human Immunodeficiency Virus / Acquired Immunodeficiency Syndrome
USAID: United States Agency for International Development
CI: Confidence interval.
IQR: Inter-quartile range
SD: Standard Deviation
